# Identification of IS*Myo2,* a novel insertion sequence element of IS*21* family and its diagnostic potential for detection of *Mycobacterium yongonense*

**DOI:** 10.1186/s12864-015-1978-2

**Published:** 2015-10-15

**Authors:** Byoung-Jun Kim, Kijeong Kim, Bo-Ram Kim, Yoon-Hoh Kook, Bum-Joon Kim

**Affiliations:** Department of Biomedical Sciences, Microbiology and Immunology, Cancer Research Institute, and Institute of Endemic Diseases, Seoul National University Medical Research Center (SNUMRC), Seoul National University College of Medicine, Seoul, Republic of Korea; Department of Microbiology, Chung-Ang University College of Medicine, Seoul, Republic of Korea; Department of Microbiology and Immunology, Liver Research Institute and Cancer Research Institute, College of Medicine, Seoul National University, 28 Yongon-dong, Chongno-gu, Seoul, 110-799 Korea

**Keywords:** *Mycobacterium yongonense*, Insertion sequence (IS) element, IS*Myo2*, Real-time PCR, Diagnostic marker

## Abstract

**Background:**

*Mycobacterium yongonense,* as a novel member of the *M. avium* complex (MAC), was recently reported to be isolated from human specimens in South Korea and Italy. Due to its close relatedness to other MAC members, particularly *M. intracellulare* in taxonomic aspects, the development of a novel diagnostic method for its specific detection is necessary for clinical or epidemiologic purposes.

**Methods:**

Using the *Mycobacterium yongonense* genome information, we have identified a novel IS-element, IS*Myo2*. Targeting the IS*Myo2* sequence, we developed a real-time PCR method and applied the technique to Mycobacterial genomic DNA.

**Results:**

To identify proper nucleic acid targets for the diagnosis, comparisons of all insertion sequence (IS) elements of 3 *M. intracellulare* and 3 *M. yongonense* strains, whose complete genome sequences we reported recently, led to the selection of a novel target gene, the *M. yongonense*-specific IS element, IS*Myo2* (2,387 bp), belonging to the IS*21* family. Next, we developed a real-time PCR method using SYBR green I for *M. yongonense*-specific detection targeting IS*Myo2*, producing a 338-bp amplicon. When this assay was applied to 28 *Mycobacterium* reference strains and 63 MAC clinical isolates, it produced amplicons in only the 6 *M. yongonense* strains, showing a sensitivity of 100 fg of genomic DNA, suggesting its feasibility as a diagnostic method for *M. yongonense* strains*.*

**Conclusions:**

We identified a novel IS*Myo2* IS element belonging to the IS*21* family specific to *M. yongonense* strains via genome analysis, and a real-time PCR method based on its sequences was developed.

**Electronic supplementary material:**

The online version of this article (doi:10.1186/s12864-015-1978-2) contains supplementary material, which is available to authorized users.

## Background

Members of the *Mycobacterium avium* complex (MAC), which are responsible for opportunistic infections, particularly in AIDS patients, are the most important nontuberculous mycobacteria (NTM) in clinical or epidemiological contexts. Traditionally, the MAC includes two species, *M. avium* and *M. intracellulare* [1–3]. In addition to these 2 species, recent advances in molecular taxonomy have fuelled the identification of novel species within the MAC [4–9].

Among these species, our group introduced the novel species *Mycobacterium yongonense*, which is phylogenetically related to *M. intracellulare* and was isolated from a Korean patient with pulmonary symptoms [10]. Notably, the RNA polymerase β subunit gene (*rpoB*) sequence of *M. yongonense* is identical to that of *M. parascrofulaceum*, a distantly related scotochromogen, suggesting acquisition of the *rpoB* gene via a potential lateral gene transfer (LGT) event [11–13]. Recently, Tortoli et al. reported pulmonary disease caused by *M. yongonense* strains isolated from patients in Italy. However, this strain notably harbors *rpoB* sequences almost identical to those of *M. intracellulare* but not to those of *M. parascrofulaceum,* suggesting the possibility of the existence of another group of *M. yongonense* strains that were not subject to the LGT event involving the *rpoB* gene from *M. parascrofulaceum* [14]. Furthermore, the potential of its misidentification has recently been proposed [15]. Therefore, the development of a novel diagnostic method for the precise identification of clinical strains of *M. yongonense* is necessary.

Insertion sequence (IS) elements have several traits of great interest in relation to epidemiological evaluations, taxonomic studies and diagnostic purposes. Depending on the degree of mobility and the copy number of IS elements, DNA fingerprints based on Southern blotting and hybridization can be used to infer strain relatedness [16]. For mycobacterial IS elements, it has generally been accepted that genetic rearrangement due to their insertion may frequently be limited to the species or subspecies level [17–20]. This specificity has led to the use of IS elements as markers for mycobacterial diagnosis, such as IS*6110* for the detection of *M. tuberculosis* [19], or IS*900* for the detection of *M. paratuberculosis* [21].

The aim of the present study is to develop a novel real-time PCR method targeting IS elements for the specific detection of *M. yongonense*. To this end, we first sought to identify the most appropriate IS element for use as a diagnostic target via comparison of the entire IS elements of 3 strains of *M. intracellulare* [ATCC 13950^T^ (NC_016946), MOTT-02 (NC_016947), and MOTT-64 (NC_016948)] and 3 strains of *M. yongonense* [DSM 45126^T^ (NC_020275), MOTT-H4Y (AKIG00000000) and MOTT-36Y (NC_017904)], whose complete genome sequences were recently reported by our group [12, 22–26]. Firstly, the two *M. yongonense* strains, MOTT-H4Y and MOTT-36Y had been identified as *M. intracellulare* INT 5 group [27]. However, our complete genome based phylogenetic analysis proved they belonged into *M. yongonense* rather than *M. intracellulare* (data not shown).

## Results

### Characterization of the IS*Myo2* IS element specific to *M. yongonense*

To select IS elements specific to *M. yongonense* strains for the diagnosis of *M. yongonense*, we compared the distributions of IS elements/transposase sequences between 3 *M. yongonense* strains (DSM 45126^T^, MOTT-36Y, and MOTT-H4Y) and 4 other MAC strains (*M. avium* 104, 3 strains of *M. intracellulare*: ATCC 13950, MOTT-02*,* and MOTT-64) via analysis of the seven retrieved mycobacterial genomes (Table 1). We identified a total of 56, 40 and 53 IS elements in *M. yongonense* DSM 45126^T^, *M. yongonense* MOTT-36Y, and *M. yongonense* MOTT-H4Y, respectively, using the IS finder program (Additional file 1). In the case of *M. yongonense* DSM 45126^T^, 12 types of IS families (IS*5*, IS*21*, IS*30*, IS*110*, IS*256*, IS*481*, IS*607*, IS*630*, IS*1380*, IS*1634*, IS*L3*, and IS*NCY*) were identified in the genome. Through comparison of the distributions of IS elements among the 7 retrieved mycobacterial genome sequences, seven IS elements (IS*Myo2*, IS*5376*, IS*Mysp3*, IS*Acl1*, IS*Mch6*, IS*Mav2*, and IS*1602*) were identified which found in only the genomes of *M. yongonense* strains. Among these IS elements, we finally targeted an IS*Mt2*- like IS element belonging to the IS*21* family, designated IS*Myo2*, which was found in only the 3 *M. yongonense* strains*,* and not in other examined strains (Additional file 2).

**Table 1:** Genome sequences used in this study

Strains	GenBank No.	Genome size (bp)	G + C ratio	CDS	tRNA	INT-group
*M. intracellulare* ATCC 13950^T^	NC_016946	5,402,402	68.10	5,145	47	INT2
*M. intracellulare* MOTT-02	NC_016947	5,409,696	68.10	5,151	47	INT2
*M. intracellulare* MOTT-64	NC_016948	5,501,090	68.07	5,251	46	INT1
*M. yongonense* DSM 45126^T^	NC_020275	5,521,023	67.95	5,147	47	INT5
*M. yongonense* MOTT-36Y	NC_017904	5,613,626	67.91	5,128	46	INT5
*M. yongonense* MOTT-H4Y	AKIG00000000	5,443,025	68.08	5,020	48	INT5
*M. avium* 104	NC_008595	5,475,491	68.99	5,120	46	-

**Fig. 1:**
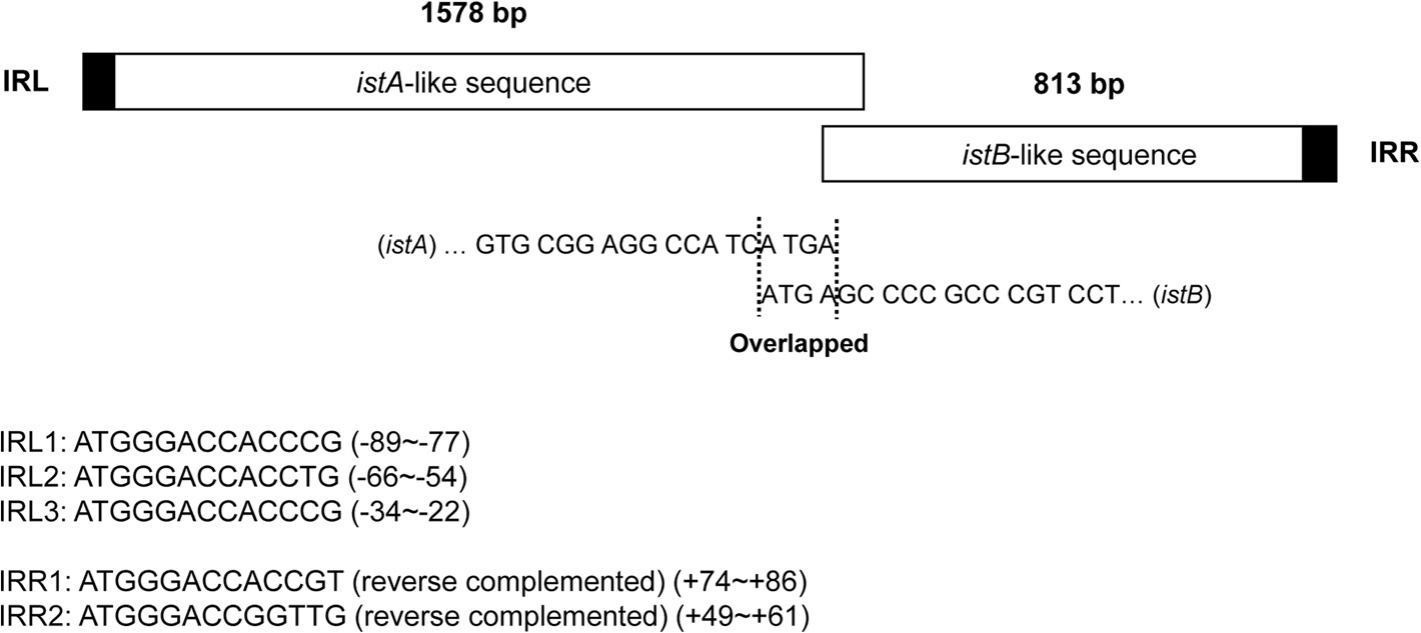
Schematic representation of the sequence of the *M. yongonense*-specific IS elements IS*Myo2*. For the *istA*-like and *istB*-like sequences, the stop and start codons overlap. Three left inverted repeat (IRL) sequences were found upstream of the *istA*-like sequence, and two right inverted repeat (IRR) sequences were found downstream of *istB*-like sequence

As shown for other IS elements of the IS*21* family [28], the *M. yongonense*-specific IS elements also exhibit two consecutive open reading frames: a long upstream frame, designated *istA* (1,578-bp), and a shorter downstream frame, designated *istB* (813-bp). *istA*- and *istB*-like sequences overlap between the stop codon of *istA* and the start codon of *istB*. Additionally, upstream and downstream of the two overlapping ORFs, there are three left inverted repeat (IRL) sequences (IRL1: ATGGGACCACCCG, IRL2: ATGGGACCACCTG, IRL3: ATGGGACCACCCG) and two right inverted repeat (IRR) sequences (IRR1: ATGGGACCACCGT, IRR2: ATGGGACCGGTTG) (Fig. 1).

A BLAST database search at the protein level revealed that *istA*- and *istB*- like sequences from *Saccharopolyspora spinosa* show sequence identities of 76.4 % (396/518) and 76.4 % (204/267), respectively, with those of IS*Myo2*. However, no mycobacterial IS elements matching IS*Myo2* were found (data not shown). Based on these findings, the IS element identified in the *M. yongonense* strains was considered a putative novel IS element belonging to the IS*21* family and was designated IS*Myo2* according to the nomenclature suggested by Mahillon and Chandler [29], and its GenBank accession No. is KP861986.

The copy numbers of IS*Myo2* in the genomes of the 3 *M. yongonense* strains, DSM 45126^T^, MOTT-36Y, and MOTT-H4Y, were 6, 5 and 4, respectively (Additional files 2 and 3). Exceptionally, in the genome of *M. yongonense* MOTT-36Y, a copy of IS*Myo2* (W7S_12150) was identified, but there was no stop codon between the *istA*- and *istB*-like sequences. Additionally, in the case of *M. yongonense* MOTT-H4Y, a copy of IS*Myo2* included only an *istB*-like sequence (W7U_06705), and no *istA*-like sequences (Additional file 3).

**Fig. 2:**
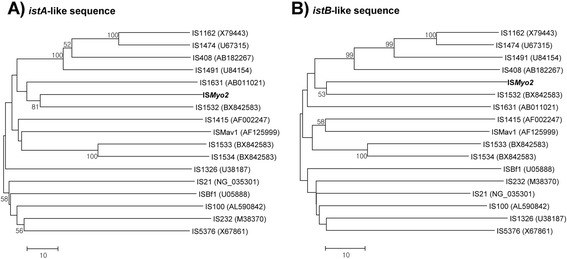
Phylogenetic trees based on (**a**) *istA*-like sequences and (**b**) *istB*-like sequences from *M. yongonense* DSM 45126^T^ and 16 other bacteria. The trees were constructed using the neighbor-joining method. Bootstrap values were calculated from 1,000 replications, and values of <50 are not indicated in the trees. The bars indicate the numbers of substitutions per amino acid position

For comparison with other IS elements related to the IS*21* family, 16 additional IS element sequences were retrieved from the GenBank database and compared with the *istA*- and *istB*-like sequences of IS*Myo2*. IS*Myo2* clustered together with IS*1532* from *M. tuberculosis* for two ORFs, though they showed low sequence similarity at the amino acid level (33.5 % for the *istA* sequence and 34.3 % for the *istB* sequence) (Fig. 2).

The phylogenetic tree based on the *istA*- and *istB*-like sequences from the *M. yongonense* strains showed the presence of four different groups (Additional file 4). In the case of *M. yongonense* DSM 45126^T^, the observed IS*Myo2* sequences were highly conserved in the genome. However, those of *M. yongonense* MOTT-36Y or MOTT-H4Y showed variations in the genomes. For the *istA* sequence (1,578 bp), the sequence homologies between the 6 alleles of *M. yongonense* DSM 45126^T^, the 5 alleles of *M. yongonense* MOTT-36Y, and the 4 alleles of *M. yongonense* MOTT-H4Y ranged from 99.7 to 100.0 %, from 75.6 to 100.0 %, and from 81.1 to 99.9 %, respectively. For the *istB* sequence (813 bp), the sequence homologies between the 6 alleles of *M. yongonense* DSM 45126^T^, the 5 alleles of *M. yongonense* MOTT-36Y, and the 4 alleles of *M. yongonense* MOTT-H4Y were 100 %, from 79.2 to 100.0 %, and from 84.7 to 100.0 %, respectively.

### Development of a real-time PCR assay targeting IS*Myo2* for the detection of *M. yongonense* strains

**Fig. 3:**
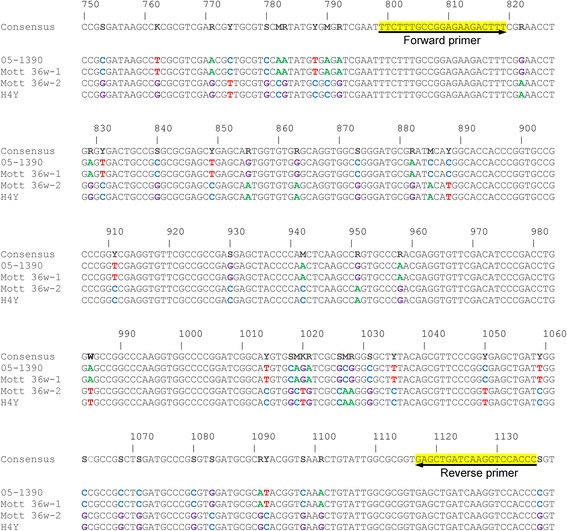
Primers designed for the identification of *Myocbacterium yongonense* on the basis of IS*Myo2* sequence alignment for *M. yongonense* strains. Arrows indicate the primer positions. The numbers indicate the nucleotide positions in the *istA*-like sequence of IS*Myo2* of *M. yongonense*. Boldface bases denote the bases that differ from those in the consensus sequence. The strains included in this analysis were as follows: *M. yongonense* DSM 45126^T^; *M. yongonense* MOTT-36Y; and *M. yongonense* MOTT-H4Y

To develop appropriate primer sets based on IS*Myo2* sequences for the specific amplification of *M. yongonense* strains, IS*Myo2* sequences from a total of 15 copies from the genomes of 3 *M. yongonense* strains were compared. Finally, we designed a primer set targeting sequences that are conserved in all 15 copies of IS*Myo2*, producing 338-bp amplicons (from the 799^th^ to the 1136^th^ nucleotide of the IS*Myo2* sequence of *M. yongonense* DSM 45126^T^ ) (Fig. 3).

### Specificity of the diagnostic assays

The specificity of the real-time PCR assay developed for the identification of *M. yongonenese* was tested in 28 reference strains of mycobacteria via Cq and melting curve analyses. Three *M. yongonense* strains (DSM 45126^T^, MOTT-36Y, and MOTT-H4Y) were specifically identified through measurement of Cq and *T*_*m*_ (~93 °C), whereas none of the 25 other reference strains showed any detectable Cqs or melting temperatures (Additional file 5, Fig. 4). The real-time PCR assay was then applied to 63 clinical isolates, including a number of MAC species; again, only the 3 *M. yongonense* strains were identified, and not the 60 other clinical MAC isolates (*M. intracellulare* INT-1: 35 strains, *M. intracellulare* INT-2: 16 strains, *M. intracellulare* INT-3: 1 strain, *M. avium*: 8 strains and *M. chimaera*: 1 strain), based on the measurement of Cqs and specific *T*_*m*_s (~93 °C) (Additional files 6, 7A and B). Agarose gel electrophoresis analysis of the real-time PCR products of 6 *M. yongonense* strains (3 reference and 3 clinical strains) revealed a single electrophoretic band of the predicted size (338 bp) (Additional file 7C).

**Fig. 4:**
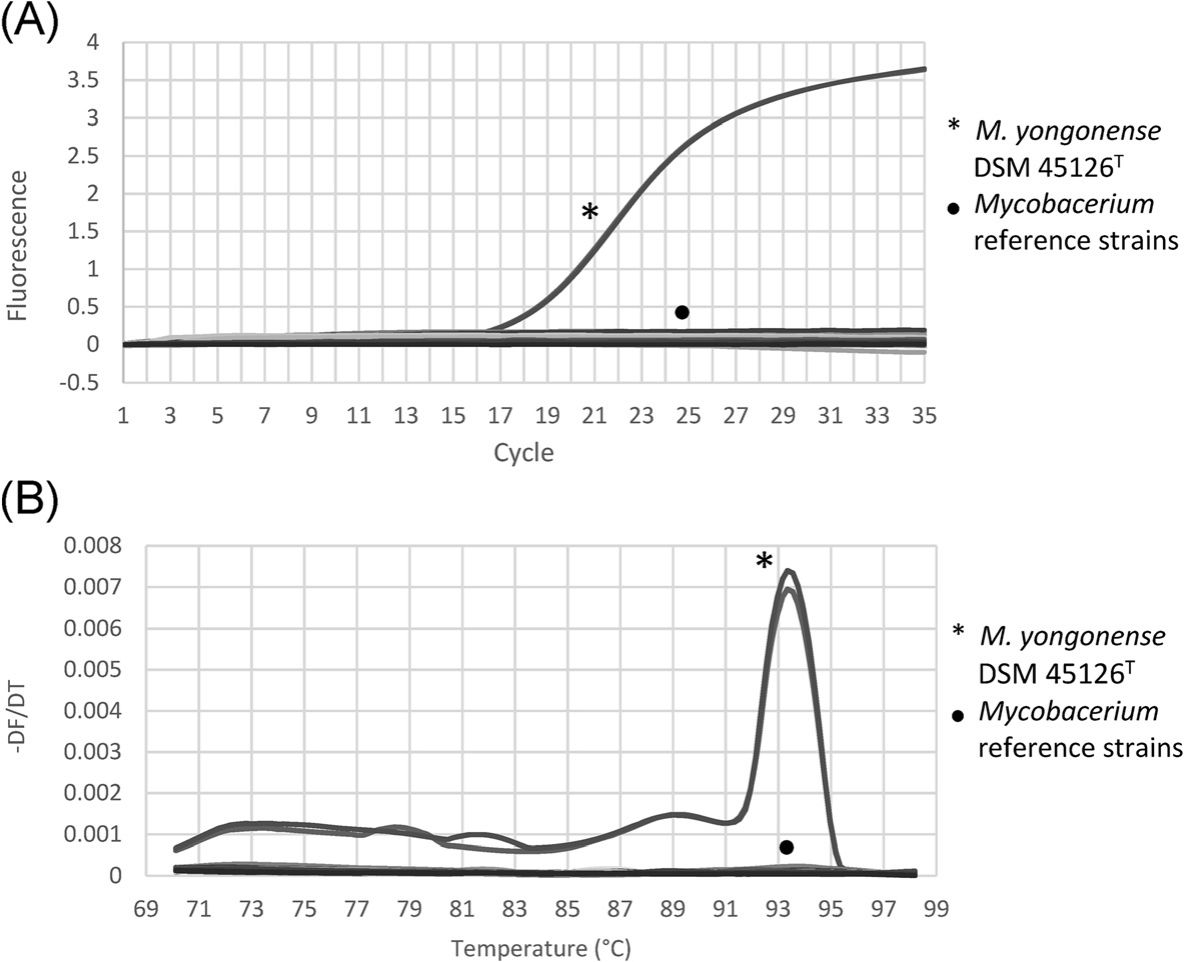
Specificity test for the real-time PCR assay developed for the identification of *M. yongonense* with using 28 reference strains of *Mycobacterium* species. *M. yongonense* was specifically identified based on Cq and melting temperature measurements. The tested strains are the same as those listed in Additional file 5 and were tested in duplicate via SYBR Green I real-time PCR. **a**, amplification curves; **b**, melting curve analysis

### Sensitivity of the diagnostic assays

To determine the detection limits of the real-time PCR assay for the detection of *M. yongonense* strains, serially diluted DNA from all 6 strains of the tested *M. yongonense* (3 reference and 3 clinical strains) was subject to real-time PCR. The detection limit of the real-time PCR assay for *M. yongonense* species was 10 fg of genomic DNA in one strain (Rhu) and 0.1 pg in all of the other tested strains (Additional file 8, Fig. 5).

**Fig. 5:**
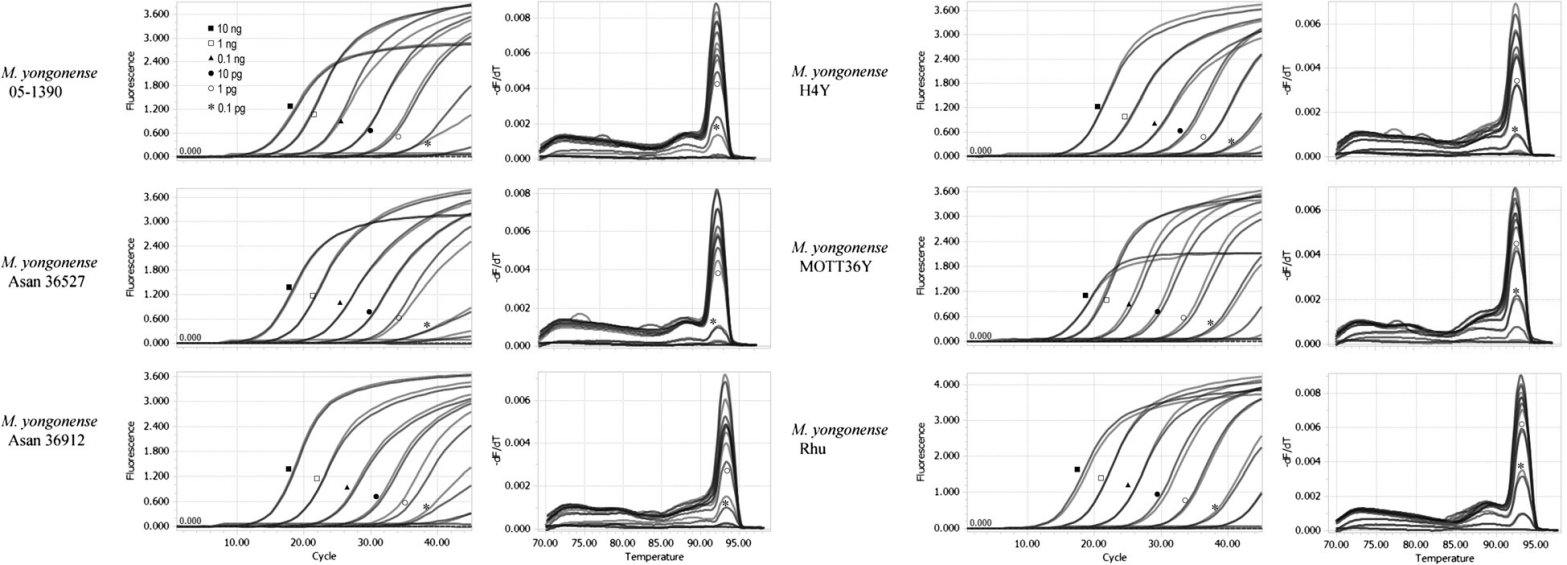
Analysis of the detection limits of the real-time PCR assay for the identification of *M. yongonense* species. All of the strains of *M. yongonense* species tested were detected, using as little as 0.1 pg of their genomic DNA

## Discussion

Among the members of the MAC complex, for the diagnosis and epidemiology of 4 *M. avium* subspecies (*M. avium* subsp. *hominissuis*, *M. avium* subsp. *avium*, *M. avium* subsp. *silvaticum* and *M. avium* subsp. *paratuberculosis*), at least four major IS elements have been widely used, namely IS*1245*, IS*1311*, IS*900* and IS*901* [30]. However, despite the increases in global clinical cases and in the species diversity of *M. intracellulare*-related strains, the description of IS elements for their diagnosis and epidemiology has rarely been reported thus far. In this study, we first introduced a novel IS element, IS*Myo2,* specific to *M. yongonense* strains, which belong to the *M. intracellulare*-related species. This element can be used for the diagnosis and epidemiology of these strains, particularly to resolve the current taxonomic need of selectively discriminating *M. yongonense* from other *M. intracellulare* related strains.

In this study, genome analysis using IS finder revealed the presence of a total of 56 IS elements and IS-like elements, consisting of at least 12 types of IS families, in the genome of *M. yongonense* DSM 45126^T^. Of these IS elements, a total of 7 were shown to be present in only the genomes of 3 *M. yongonense* strains, and were absent in the genomes of 3 *M. intracellulare* strains (Additional file 2). However, considering the copy numbers, BLAST search results, and sequence conservation between IS alleles in a genome, we finally selected IS*Myo2*, belonging to the IS*21* family, as a target IS element for the diagnosis of *M. yongonense* strains.

It is worth noting several characteristic features of IS*Myo2* that make it a useful diagnostic marker for *M. yongonense*. The first is the high specificity of IS*Myo2* for *M. yongonense*. To date, 4 types of IS*21* family members have been described in the genus *Mycobacterium*, three in *M. tuberculosis* (IS*1532*, IS*1533* and IS*1534*) [31] and one in *M. avium* (IS*Mav1*) [30]*.* Despite belonging to the IS*21* family, the IS*Myo2* element identified in this study shows a very low level of sequence homology with other IS*21* members from mycobacteria, particularly in terms of DNA sequences, suggesting the feasibility of using IS*Myo2* as a diagnostic marker for *M. yongonense.* Furthermore, the application of a real-time PCR assay targeting IS*Myo2* in reference mycobacterial strains demonstrated that the assay was specific to only *M. yongonense* strains (Fig. 4, Additional file 5). Furthermore, it did not produce any amplicons in any of the 61 examined *M. intracellulare* clinical isolates, which included diverse genotypes such as INT-1, INT-2 and INT-3, which are closely related to *M. yongonense* (Additional files 6 and 7)*.*

Second, the IS*Myo2-* targeting assay was able to detect 2 types of *M. yongonense* variants. Our genome analysis revealed that IS*Myo2* is also present in the genomes of 2 strains of *M. yongonense*, MOTT-36Y and MOTT-H4Y, which were previously designated as the *M. intracellulare* INT-5 genotype, as well as in genome of *M. yongonense* DSM 45126^T^. Although two INT-5 strains exhibit *rpoB* sequences identical to that of *M. intracellulare* but not to that of *M. parascrofulaceum*, our phylogenetic analysis based on complete genome sequences showed that these strains and *M. yongonense* DSM 45126^T^ are tightly clustered and are separated from other *M. intracellulare* strains (data not shown), suggesting that they may be members of the same species, *M. yongonense.* Our results also strongly supported the hypothesis previously put forth by Tortoli *et al*. [14] that there may be at least two variants in *M. yongonense* strains, one of which was subjected to the *rpoB* LGT event from *M. parascrofulaceum*, including strains such as *M. yongonense* DSM 45126^T^, and another, phylogenetically older variant, that was not subject to the *rpoB* LGT, including strains such as the INT-5 strains MOTT-36Y and MOTT-H4Y.

Third, the IS*Myo2* targeting assay exhibited high sensitivity in detecting *M. yongonense* strains. Our genome analysis showed that more than 4 copies of IS*Myo2* are present in *M. yongonense* genomes (Additional files 3 and 4). Furthermore, the sequence conservation between the IS*Myo2* alleles found in the *M. yongonense* genomes facilitated the development of a common primer set capable of PCR amplification of all of these alleles (Fig. 3). Thus, we successfully developed a real-time PCR assay capable of PCR amplification of all of the alleles via performing multiple sequence alignments of 15 IS*Myo2* alleles from the genomes of 3 *M. yongonense* strains. Our real-time PCR assay can detect PCR amplicons at a DNA level of 100 fg in all 6 *M. yongonense* strains (Fig. 5, Additional file 8), suggesting its feasibility as a diagnostic method for *M. yongonense* strains*.*

## Conclusions

In conclusion, we identified a novel IS*Myo2* IS element belonging to the IS*21* family that is specific to *M. yongonense* strains via genome analysis and developed a real-time PCR method based on its sequences.

## Methods

### Genome sequences used in this study

Seven mycobacterial genome sequences, from strains belonging to the *M. avium* complex (3 *M. intracellulare* strains: ATCC 13950^T^, MOTT-02, and MOTT-64; 3 *M. yongonense* strains: DSM 45126^T^, MOTT-36Y, and MOTT-H4Y; and one *M. avium* strain: *M. avium* 104) were retrieved from the GenBank database (Table 1) and used for comparative analysis of IS-elements.

### Mycobacterial strains

Twenty-eight mycobacterial reference strains (Additional file 5) and 63 clinical isolates (Additional file 6) were used in this study. Twenty-three of the 28 mycobacterial reference strains (with the exception of *M. massiliense* KCTC 19086^T^ and 3 *M. yongonense* strains, DSM 45126^T^, MOTT-H4Y, MOTT-36Y and *M. tuberculosis* ATCC 27294^T^) were provided by the Korean Institute of Tuberculosis (KIT). In the case of *M. tuberculosis* ATCC 27294^T^, genomic DNA of *M. tuberculosis* was provided from the KIT. The *M. massiliense* KCTC 19086^T^ strain was provided by the Korean Collection for Type Cultures (KCTC), and the three *M. yongonense* strains, DSM 45126^T^, MOTT-H4Y and MOTT-36Y, were from Seoul National University College of Medicine (SNUMC). No ethics approval was required for the bacterial isolates in this study.

### Analysis of insertion sequence (IS) elements

Using the sequence information from the seven mycobacterial genomes, and especially the information on annotated genes, IS elements from each genome were identified. IS elements from *M. yongonense* DSM 45126^T^ were identified using a default parameter of the IS-finder tool (http://www-is.biotoul.fr) [32] and compared with IS elements from the other six mycobacterial genome sequences using the MegAlign (DNASTAR package, http://www.dnastar.com/t-megalign.aspx) [33] and BLAST (http://blast.ncbi.nlm.nih.gov/Blast.cgi) programs. To identify terminal inverted repeat (IR) sequences, upstream and downstream sequences of the targeted *M. yongonense*-specific IS element were analyzed using the Spectral Repeat Finder tool (http://www.imtech.res.in/raghava/srf/) with default parameters except for a parameter of ‘minimum number of copies’ (we used this parameter value with 2, the default value was 5) [34].

### Phylogenetic analysis

The identified novel IS element IS*Myo2* was compared with the IS elements from 2 other *M. yongonense* strains (*M. yongonense* MOTT-36Y and *M. yongonense* MOTT-H4Y) and 16 known IS elements belonging to the IS*21* family. Amino acid or nucleotide sequences were aligned using the ClustalW method in MEGA 4 software [35]. Phylogenetic trees were constructed using the neighbor-joining method [36] and maximum composite likelihood (in the case of nucleotide sequences) or number of differences substitution models (in the case of amino acid sequences) in MEGA 4 software [35]. Bootstrap values were calculated from 1,000 replications.

### DNA extraction

To prepare genomic DNA from reference or clinical isolated mycobacteria, strains were cultured on the 7H10 agar plate (supplemented with OADC) for 7 to 10 days in the 37 °C, 5 % CO_2_ incubator. Chromosomal DNA was extracted from single colonies of the clinical isolates via the bead beater–phenol extraction method as described previously [37].

### Primer design

A set of primers [forward primer, 5′-TTCTTTGCCGGAGAAGACTTT-3′; reverse primer, 5′-GGGTGGACCTTGATCAGCTC-3′] was designed to produce a 338-bp IS*Myo2* amplicon (from the 799th to 1136th nucleotide in the *M. yongonense* DSM 45126^T^ IS*Myo2* sequence) from all strains of *M. yongonense* using Oligo V 6.5 (Molecular Biology Insights).

### Real-time PCR

The LightCycler 96 system was used for real-time PCR. A 10 μl reaction mixture was prepared for each sample, with the following components: 1 μl of Taq buffer (including 20 mM MgCl_2_) supplied together with Ex *Taq* HS (Takara), 0.25 μM forward primer, 0.25 μM reverse primer, 0.2 mM dNTPs, 0.7 mg/ml BSA (NEB), 0.5 × SYBR Green I (Sigma S9430), 3 % DMSO, 0.25 U of ExTaq HS, and sterile distilled water. The cycling conditions were 2 min at 95 °C and 5 s at 98 °C, followed by 35 or 45 cycles of 10 s at 98 °C, 10 s at 64 °C and 40 s at 72 °C (single acquisition of fluorescence signals). Melting curve analysis was performed as follows: 10 s at 98 °C and 1 min at 70 °C, after which the temperature was increased from 70 °C to 98 °C at a temperature transition rate of 0.2 °C/s, with continuous acquisition of the fluorescence signal. Quantification cycles (C_q_s) and amplicon melting temperatures (*T*_*m*_s) were measured using LightCycler 96 system software, V1.1. The *T*_*m*_ specificity was verified via duplicate real-time PCR measurements with a panel of reference mycobacterial DNAs. A total of 63 clinical isolates were subsequently tested for the identification of *M. yongonense* species. The detection limit of the real-time PCR assay for *M. yongonense* was tested in duplicate using serially diluted genomic DNA from 10 ng to 10 fg.

### Availability of supporting data

The data sets supporting the results of this article are included within the article and its additional files (Additional files 1, 2, 3, 4, 5, 6, 7 and 8).

## Additional files

Additional file 1:
**Distributions of IS-element/transposase in** ***M. avium*** **complex strains.** (XLSX 8 kb) Additional file 2:
**Distributions of IS-elements from** ***M. yongonense*** **and other** ***M. intracellulare*** **strains.** (XLSX 10 kb) Additional file 3:
**IS** ***Myo2*** **elements in the genomes of** ***M. yongonense*** **DSM 45126** ^**T**^, ***M. yongonense*** **MOTT-36Y and** ***M. yongonense*** **MOTT-H4Y.** (XLSX 10 kb) Additional file 4:
**Phylogenetic tree based on (A)** ***istA*** **-like sequences and (B)** ***istB*** **-like sequences from** ***M. yongonense*** **DSM 45126** ^**T**^, ***M. yongonense*** **MOTT-36Y and** ***M. yongonense*** **MOTT-H4Y.** IS-elements of *M. tuberculosis* were used as an outgroup. The trees were constructed using the neighbor-joining method. The bootstrap values were calculated from 1,000 replications and <50 were not indicated. The bars indicate numbers of substitutions per nucleotide position. (PDF 76 kb) Additional file 5:
**Mycobacterial reference strains used in the present study and specificity of real-time PCR for the detection of the insertion sequence specific to** ***Mycobacterium yongonense.*** (XLSX 11 kb) Additional file 6:
**Identification of the insertion sequence specific to** ***M. yongonense*** **among clinical isolates by real-time PCR.** (XLSX 12 kb) Additional file 7:
**Real-time PCR identification of** ***M. yongonense*** **from clinical isolates of** ***Mycobacterium*** **species.** All the *M. yongonense* species among the clinical isolates tested were specifically identified by measurement of their Cqs and melting temperatures. The clinical isolates tested are the same ones listed in Additional file 2 and were tested in duplicate by SYBR Green I real-time PCR. (A) amplication curves. (B) melting curve analysis. (C) agarose gel electrophoresis analysis of real-time PCR products. M, 100 bp DNA marker; 1, *M. yongonense* DSM 45126^T^; 2, *M. yongonense* Asan 36527; 3, *M. yongonense* Asan 36912; 4, *M. yongonense* MOTT-H4Y; 5, *M. yongonense* MOTT-36Y; 6, *M. yongonense* Rhu; 7, negative control. (PDF 250 kb) Additional file 8:
**Limit of detection of real-time PCR for the identification of the insertion sequence specific to** ***M. yongonense.*** (XLSX 10 kb)

## Abbreviations

BLAST: bagic local alignment search tool
BSA: bovine serum albumin
Cq: quantification cycle
dNTP: deoxynucleotide triphosphate
fg: femto gram
IR: inverted repeat
IRL: left inverted repeat
IRR: right inverted repeat
IS element: insertion sequence element
KCTC: Korean Collection for Type Culture
KIT: Korean Institute of Tuberculosis
LGT: lateral gene transfer
MAC: *Mycobacterium avium* complex
ng: nano gram
NTM: nontuberculous mycobacteria
ORF: open reading frame
PCR: polymerase chain reaction
pg: pico gram
*rpoB*: RNA polymerase β subunit gene
SNUMC: Seoul National University College of Medicine
