# Supplementary figures and images for: Identification of ISMyo2, a novel insertion sequence element of IS21 family and its diagnostic potential for detection of Mycobacterium yongonense
Source: BMC Genomics. 2015 Oct 15;16:794. doi: 10.1186/s12864-015-1978-2 (PMC4608216; doi:10.1186/s12864-015-1978-2)

**(A) *istA*-like sequence**

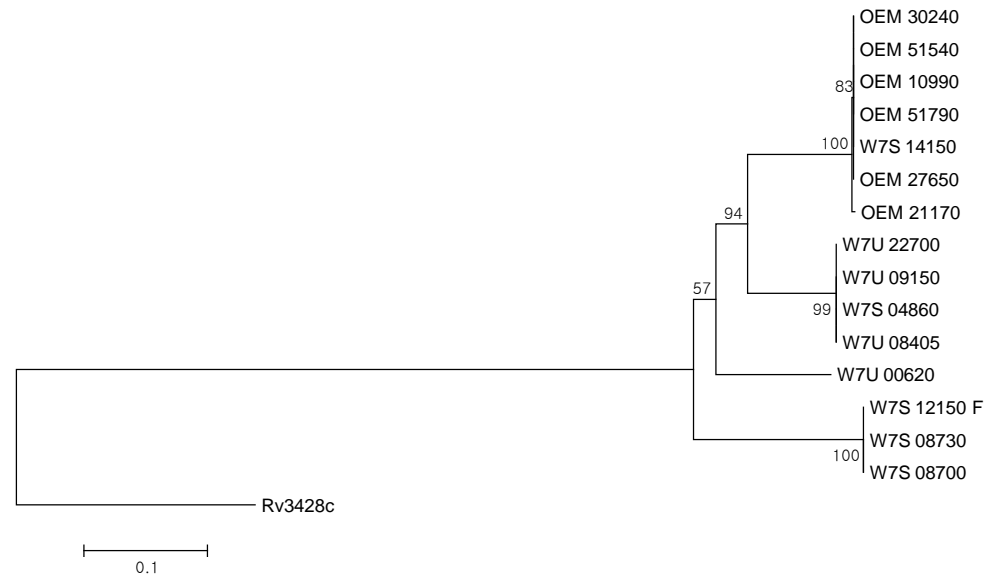

**(B) *istB*-like sequence**

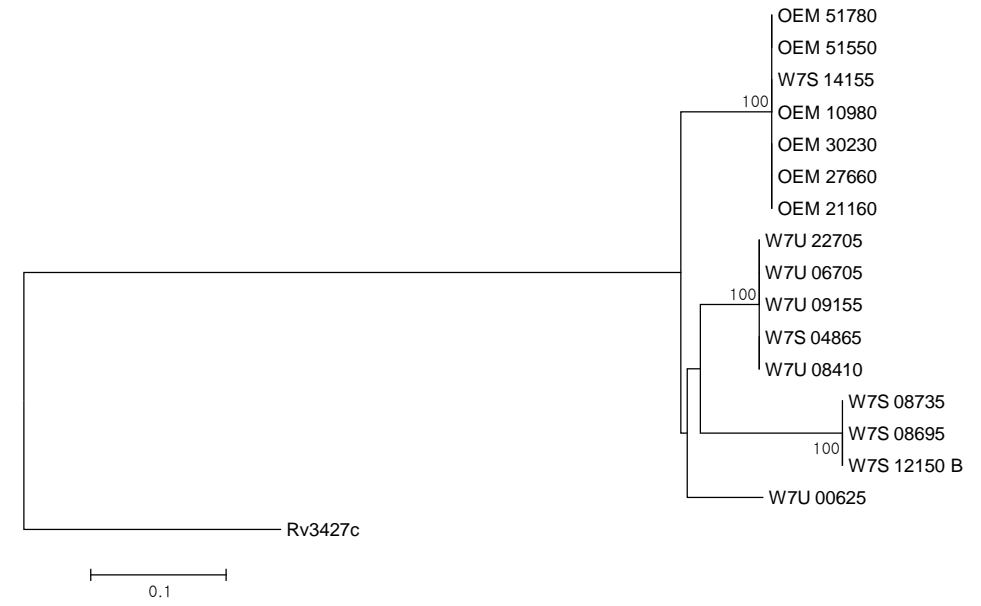

Supplement: Additional file 4: — Phylogenetic tree based on (A) istA -like sequences and (B) istB -like sequences from M. yongonense DSM 45126 T, M. yongonense MOTT-36Y and M. yongonense MOTT-H4Y. IS-elements of M. tuberculosis were used as an outgroup. The trees were constructed using the neighbor-joining method. The bootstrap values were calculated from 1,000 replications and <50 were not indicated. The bars indicate numbers of substitutions per nucleotide position. (PDF 76 kb) [file 12864_2015_1978_MOESM4_ESM.pdf]
