# Supplementary material for: Identification of ISMyo2, a novel insertion sequence element of IS21 family and its diagnostic potential for detection of Mycobacterium yongonense
Source: BMC Genomics. 2015 Oct 15;16:794. doi: 10.1186/s12864-015-1978-2 (PMC4608216; doi:10.1186/s12864-015-1978-2)
Supplement: Additional file 7: — Real-time PCR identification of M. yongonense from clinical isolates of Mycobacterium species. All the M. yongonense species among the clinical isolates tested were specifically identified by measurement of their Cqs and melting temperatures. The clinical isolates tested are the same ones listed in Additional file 2 and were tested in duplicate by SYBR Green I real-time PCR. (A) amplication curves. (B) melting curve analysis. (C) agarose gel electrophoresis analysis of real-time PCR products. M, 100 bp DNA marker; 1, M. yongonense DSM 45126T; 2, M. yongonense Asan 36527; 3, M. yongonense Asan 36912; 4, M. yongonense MOTT-H4Y; 5, M. yongonense MOTT-36Y; 6, M. yongonense Rhu; 7, negative control. (PDF 250 kb) [file 12864_2015_1978_MOESM7_ESM.pdf]

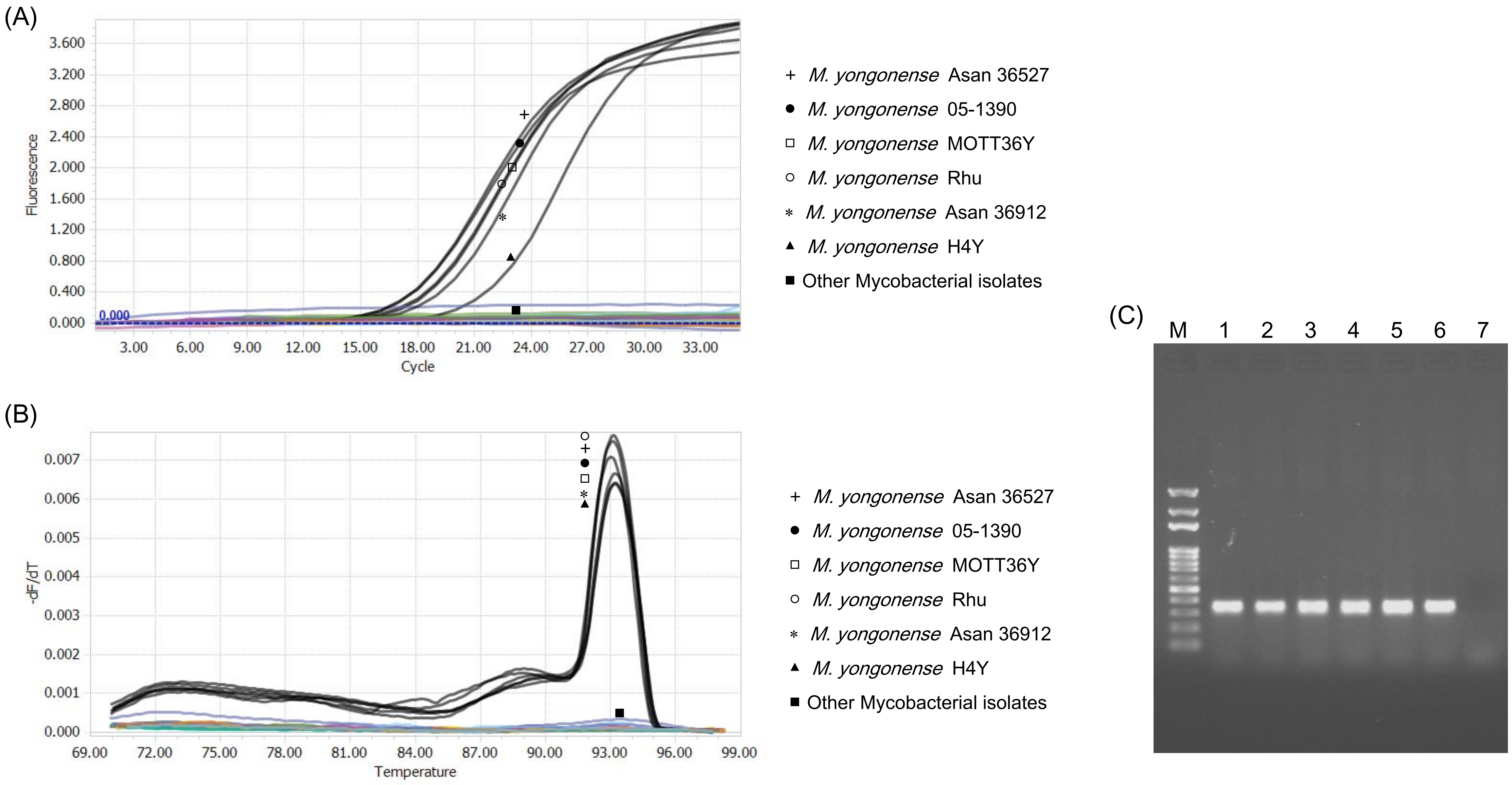

(A)
3.600
3.200
2.800
2.400
2.000
1.600
1.200
0.800
0.400
0.000
Fluorescence
0.000
3.00
6.00
9.00
12.00
15.00
18.00
21.00
24.00
27.00
30.00
33.00
Cycle
+ M. yongonense Asan 36527
● M. yongonense 05-1390
□ M. yongonense MOTT36Y
○ M. yongonense Rhu
* M. yongonense Asan 36912
▲ M. yongonense H4Y
■ Other Mycobacterial isolates
(B)
0.007
0.006
0.005
0.004
0.003
0.002
0.001
0.000
-dF/dT
69.00
72.00
75.00
78.00
81.00
84.00
87.00
90.00
93.00
96.00
99.00
Temperature
+ M. yongonense Asan 36527
● M. yongonense 05-1390
□ M. yongonense MOTT36Y
○ M. yongonense Rhu
* M. yongonense Asan 36912
▲ M. yongonense H4Y
■ Other Mycobacterial isolates
(C)
M
1
2
3
4
5
6
7
